# Identification of potential drug targets for insomnia by Mendelian randomization analysis based on plasma proteomics

**DOI:** 10.3389/fneur.2024.1380321

**Published:** 2024-04-25

**Authors:** Ni Yang, Liangyuan Shi, Pengfei Xu, Fang Ren, Shimeng Lv, Chunlin Li, Xianghua Qi

**Affiliations:** ^1^Department of First Clinical Medical College, Shandong University of Traditional Chinese Medicine, Jinan, China; ^2^Qingdao Traditional Chinese Medicine Hospital (Qingdao Hiser Hospital) Qingdao Hiser Hospital Affiliated of Qingdao University, Qingdao, China; ^3^Department of Laboratory, Jimo District Qingdao Hospital of Traditional Chinese Medicine, Qingdao, China; ^4^Affiliated Hospital of Shandong University of Traditional Chinese Medicine, Jinan, China

**Keywords:** insomnia, Mendelian randomization, drug target, plasma proteomics, protein quantitative trait loci

## Abstract

**Introduction:**

Insomnia, a common clinical disorder, significantly impacts the physical and mental well-being of patients. Currently, available hypnotic medications are unsatisfactory due to adverse reactions and dependency, necessitating the identification of new drug targets for the treatment of insomnia.

**Methods:**

In this study, we utilized 734 plasma proteins as genetic instruments obtained from genome-wide association studies to conduct a Mendelian randomization analysis, with insomnia as the outcome variable, to identify potential drug targets for insomnia. Additionally, we validated our results externally using other datasets. Sensitivity analyses entailed reverse Mendelian randomization analysis, Bayesian co-localization analysis, and phenotype scanning. Furthermore, we constructed a protein-protein interaction network to elucidate potential correlations between the identified proteins and existing targets.

**Results:**

Mendelian randomization analysis indicated that elevated levels of TGFBI (OR = 1.01; 95% CI, 1.01–1.02) and PAM ((OR = 1.01; 95% CI, 1.01–1.02) in plasma are associated with an increased risk of insomnia, with external validation supporting these findings. Moreover, there was no evidence of reverse causality for these two proteins. Co-localization analysis confirmed that PAM (coloc.abf-PPH4 = 0.823) shared the same variant with insomnia, further substantiating its potential role as a therapeutic target. There are interactive relationships between the potential proteins and existing targets of insomnia.

**Conclusion:**

Overall, our findings suggested that elevated plasma levels of TGFBI and PAM are connected with an increased risk of insomnia and might be promising therapeutic targets, particularly PAM. However, further exploration is necessary to fully understand the underlying mechanisms involved.

## 1 Introduction

Insomnia is a prevalent clinical disorder characterized by difficulties in falling asleep or maintaining sleep at night, as well as daytime fatigue, decreased attention and memory, and emotional disorders, among others (1). According to population data, approximately 30–40% of adults report at least one symptom of nocturnal insomnia, with 10–15% experiencing adverse effects such as fatigue during the day (2–5). Insomnia impacts the patient’s quality of life and exhibits a causal relationship with physical and mental illnesses, including cardiovascular disease (6), anxiety (7), depression (8), and even cancer (9). Therefore, it is imperative to focus on the treatment of insomnia.

Currently, the primary classes of drugs used for treating insomnia include benzodiazepine receptor agonists, benzodiazepines, dual orexin receptor antagonists, sedative antidepressants, melatonin, melatonin receptor agonists, and barbiturates (10). However, these drugs may be associated with adverse reactions like headache, dizziness, nausea, and drowsiness. Additionally, long-term use can lead to dependence, resulting in rebound insomnia or withdrawal symptoms after discontinuation of the medication (11). The search for effective and less adverse drugs to treat insomnia remains a pressing issue in clinical practice.

Plasma proteins play a pivotal role in numerous biological processes and are the essential source of drug targets (12). Genetic associations linking protein drug targets to diseases can significantly increase the success rate of clinical development (13). Whole genome association studies (GWAS) have yielded critical insights into complex diseases, which are anticipated to facilitate the discovery of novel drug targets and therapeutic opportunities (14). Mendelian randomization (MR) employs single nucleotide polymorphisms (SNPs) from GWAS as genetic tools to evaluate the causal impact of exposure on outcomes, thereby promoting the recognition of possible therapeutic targets for numerous diseases (15).

**Figure 1:**
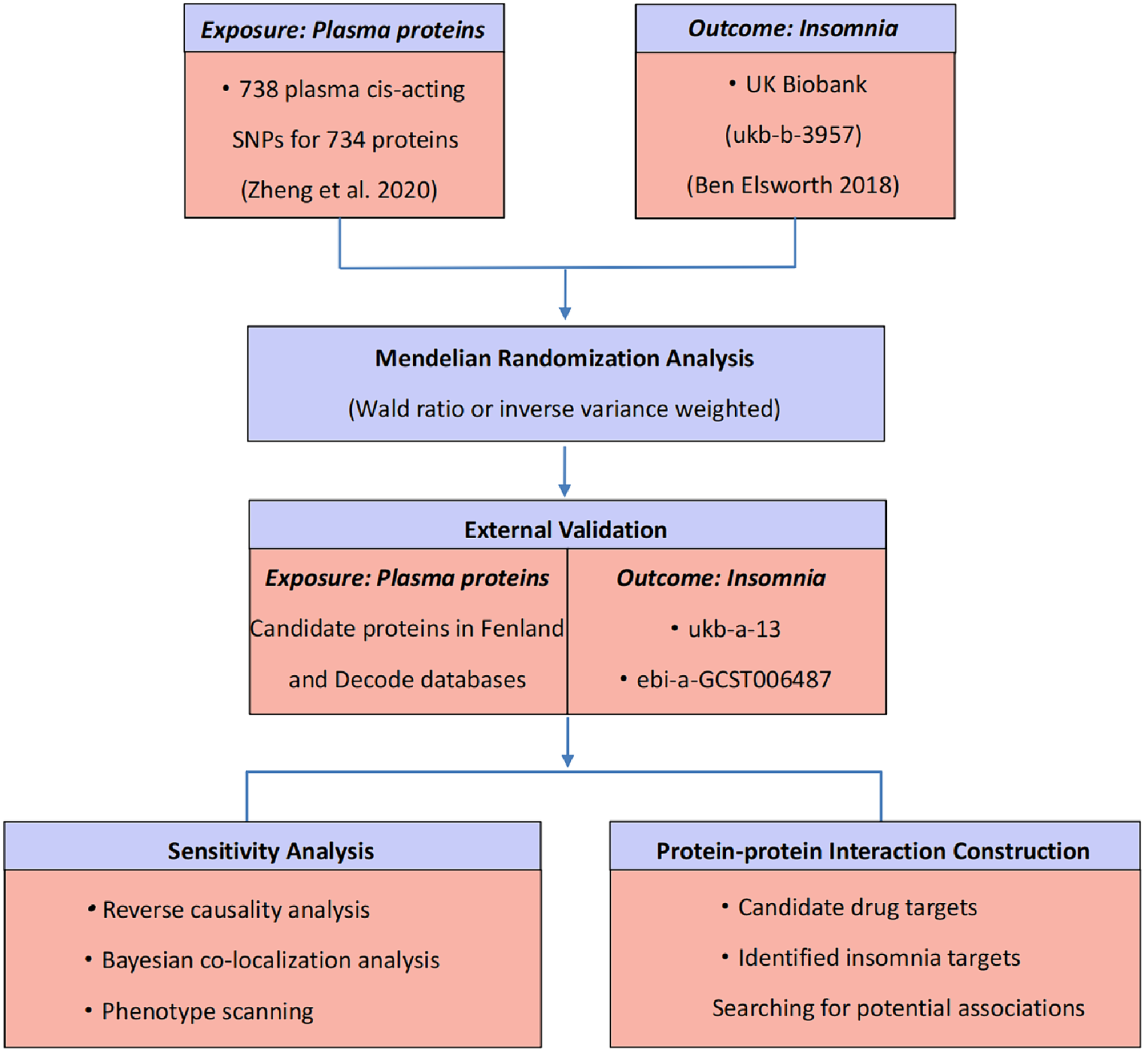
Study design for identification of potential drug targets for insomnia based on plasma proteomics.

Our study was to detect plasma proteins that could serve as potential drug targets for insomnia. We utilized insomnia GWAS data and plasma pQTL data to determine prospective pathogenic plasma proteins and validated these findings through external GWAS. Subsequently, we conducted further validation by reverse causality test, Bayesian co-localization analysis, as well as phenotype scanning. Finally, we investigated the potential mechanisms of action by constructing an interaction network involving known pathogenic targets. The research design is depicted in Figure 1, and the databases and analysis websites involved in the study are illustrated in Supplementary Table S1.

## 2 Materials and methods

### 2.1 Data sources

#### 2.1.1 Plasma cis-acting protein quantitative trait loci

The initial analysis data of plasma cis-acting protein quantitative trait loci (pQTLs) were acquired from Zheng et al.’s research (12), which included five genome-wide association studies (GWAS) (16–20). To ensure data reliability, we used the original files as the reference. Two additional datasets were employed for external validation, sourced from the deCODE (21) and Fenland (22) databases. These datasets included measurements of plasma proteins in 10,708 and 35,559 individuals, with a total of 4,775 and 4,907 proteins, respectively. The inclusion criteria for the cis-acting pQTLs were as follows: (1) they reached genome-wide significance (*p* < 5 × 10^−8^), (2) they were situated beyond the major histocompatibility complex region (chr6, 26–34 Mb), and (3) they demonstrated independence [linkage disequilibrium (LD) clumping r^2^ < 0.001]. In cases where information such as the effect allele frequency was missing in the pQTL GWAS, the matched human genome build was referenced to complete the data.

#### 2.1.2 GWAS data of insomnia

The summary data of insomnia for the basic analysis were extracted from the IEU Open GWAS (id: ukb-b-3957), which included 462,341 European participants. These data were derived variables from the UK Biobank and were processed by a GWAS pipeline. The summary data for insomnia in the validation set were obtained from the Neale Lab (id: ukb-a-13) and from a genome-wide association analysis of insomnia complaints (id: ebi-a-GCST006487) (23), with 336,965 and 59,367 Europeans, separately.

### 2.2 Statistical analysis

#### 2.2.1 MR analysis

The MR analysis was performed using plasma cis-acting pQTL as the exposure variable and insomnia as the outcome variable through the TwoSampleMR package in Rstudio 4.1.3. When there were more than one genetic instruments, we applied the inverse variance weighted (IVW) method. However, if a given protein only had one pQTL, we used the Wald ratio before conducting the heterogeneity analysis. The odds ratio (OR) for a higher risk of insomnia was presented per standard deviation (SD) increase in plasma cis-acting pQTL level. We utilized the Bonferroni correction to adjust for multiple testing in the primary analysis. The significance level was considered as *p* < 0.05/734. Subsequently, the external validation MR analysis was conducted on those recognized proteins. We selected the same SNP as well as genome-wide significant SNPs in the original research as the genetic instruments. *p* < 0.05 was considered statistically significant.

#### 2.2.2 The sensitivity analysis

##### 2.2.2.1 Reverse causality detection

To investigate potential reverse causality, we conducted bidirectional MR analysis on those proteins with statistical significance. Based on the pQTL screening criteria, we retrieved genetic instruments from the GWAS of insomnia in the preliminary analysis. The complete summary statistical data from the original study of the corresponding protein was as the outcome variable, and MR-IVW, MR Egger, weighted median, simple mode, and weighted mode were used to assess the effect. We applied Steiger filtering (24) to confirm the existence of a directional connection between proteins and insomnia. The significance level was considered as *p* < 0.05.

##### 2.2.2.2 Co-localization analysis

The Bayesian co-localization analysis was implemented through the “coloc” package to estimate the possibility of two features sharing the same causal variant. In the coloc.abf algorithm, we calculated the posterior probabilities of five mutually exclusive hypotheses (PPH0–PPH4), and a gene was defined to have gene-based co-localization evidence when PPH4 exceeds 80%. This indicated a connection between the protein and insomnia through shared variants in the region.

##### 2.2.2.3 Phenotype scanning

To explore the relationship of recognized pQTLs with other traits, we utilized “Phenoscanner” (25) for phenotype scanning by searching previous GWAS. When a SNP reached the genome-wide significant level (*p* < 5 × 10^−8^) and was related to the risk factors of insomnia among the GWAS of Europeans, it was determined pleiotropic.

#### 2.2.3 Protein–protein interaction network

We found a PPI network by searching the DrugBank and Open Targets databases and comparing them with significant proteins in the basic analysis to investigate the interactions between the target proteins. Given the limited availability of drug targets at present, we also built a PPI network by searching the GeneCards database for the top 335 targets with a relevance score greater than 1.5 of insomnia and exploring the interactions between proteins. The PPI network was established by the STRING database with a minimum interaction score of 0.4 and was visualized via Cytoscape 3.9.1.

## 3 Results

### 3.1 Screening for causal proteins in insomnia

**Figure 2:**
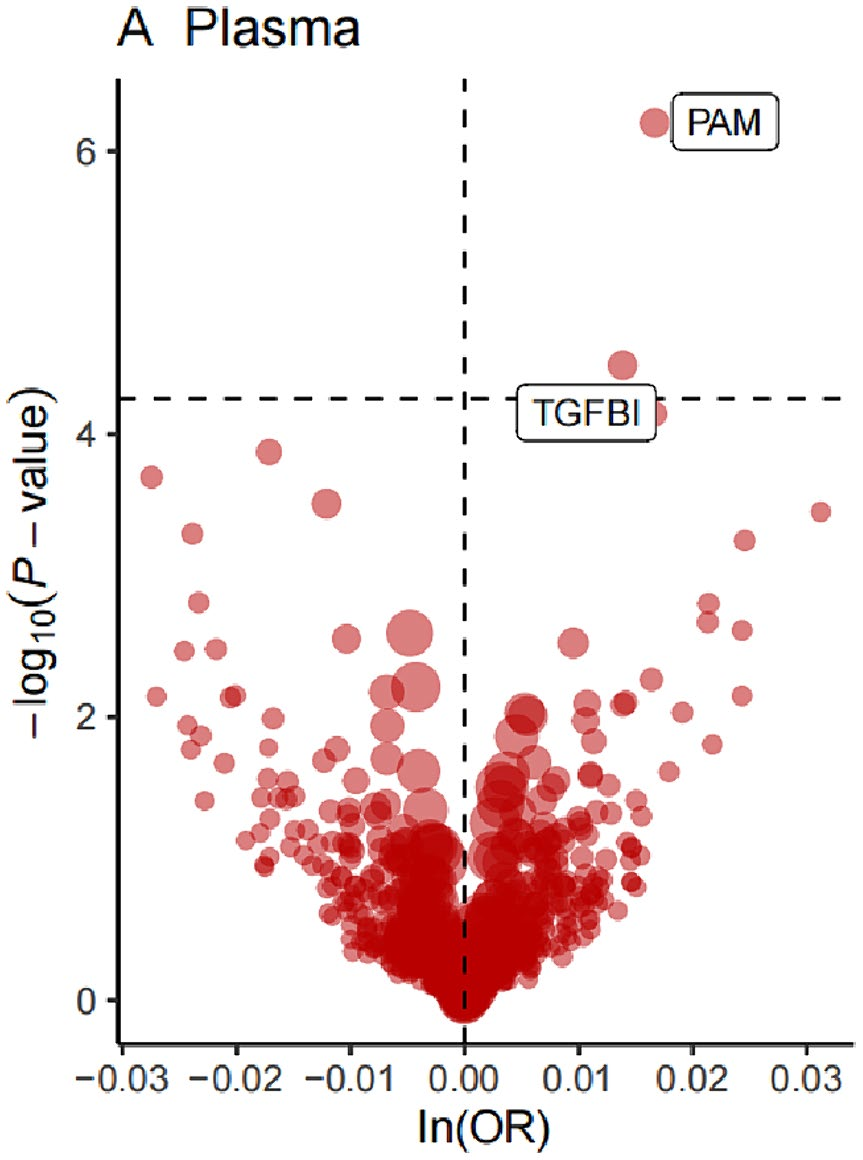
The volcano plot of the MR results for 734 plasma proteins on the risk of insomnia.

For the initial MR analysis, we included a total of 738 plasma cis-acting SNPs from 734 proteins (Supplementary Table S2) based on the screening criteria, and two protein-insomnia pairs were identified with Bonferroni significance (*p* < 0.05/734). Specifically, elevated levels of TGFBI (OR = 1.01; 95% CI, 1.01–1.02; *p* = 3.26E-05) and PAM (OR = 1.01; 95% CI, 1.01–1.02; *p* = 6.32E-07) were found to increase the risk of insomnia. The detailed results of the initial MR analysis of TGFBI and PAM are shown in Table 1. Figure 2 depicts the visualized volcano plot. Heterogeneity analysis on proteins with two or more instruments revealed that only GPC5 exhibited significant heterogeneity (*p* = 0.006). The results of the heterogeneity analysis can be found in the Supplementary Table S3.

**Table 1:** The significantly associated plasma proteins with insomnia after Bonferroni correction.

Protein	UniProt ID	SNP	Effect allele	OR (95% CI)	*p*-value	PVE	F statistics	Author
TGFBI	A0A0S2Z4Q2; Q15582	rs13159365	T	1.01 (1.01, 1.02)	3.26E-05	9.55%	348.39	Sun
PAM	P19021; O43832	rs257309	G	1.02 (1.01, 1.02)	6.32E-07	9.68%	353.68	Sun

OR, odds ratio, per standard deviation increase in plasma protein levels; PVE, proportion of variance explained.

**Figure 3:**
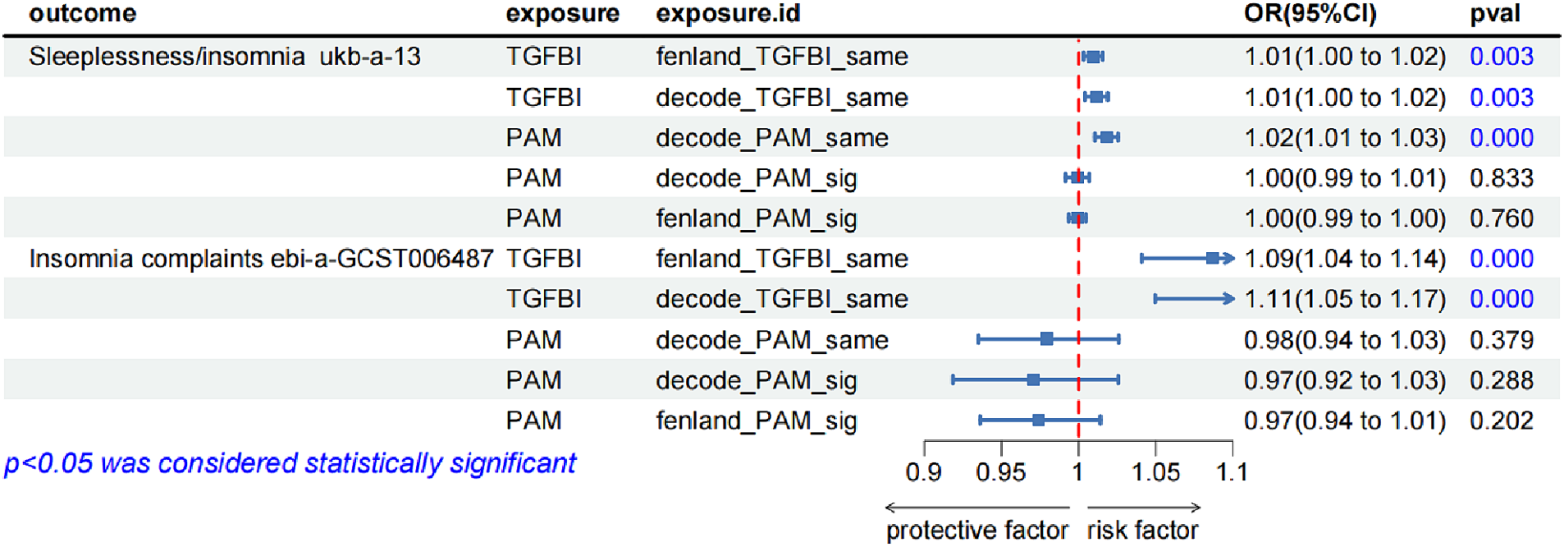
The forest diagram of the external validation analysis.

To validate the robustness of our main findings, we employed both the same-variant and significant-variant strategies in various datasets. From the deCODE database, we filtered the same variant SNPs of TGFBA and PAM as well as the significant variation of PAM. Simultaneously, in the Fenland database, we screened the same variant of TGFBI and the significant variant of PAM (Supplementary Table S4). These variants were then used for the external validation analysis of MR exposure in two insomnia cohorts. The results indicated that TGFBI was consistently identified as a pathogenic factor for insomnia in both the UK Biobank and GWAS-Catalog cohorts. On the other hand, PAM was observed to be associated with insomnia only in the UK Biobank database. Notably, we only detected causal relationships within the same variants, rather than between the significant variants. Figure 3 shows the forest diagram of the external validation analysis.

### 3.2 Sensitivity analysis for causal proteins

**Figure 4:**
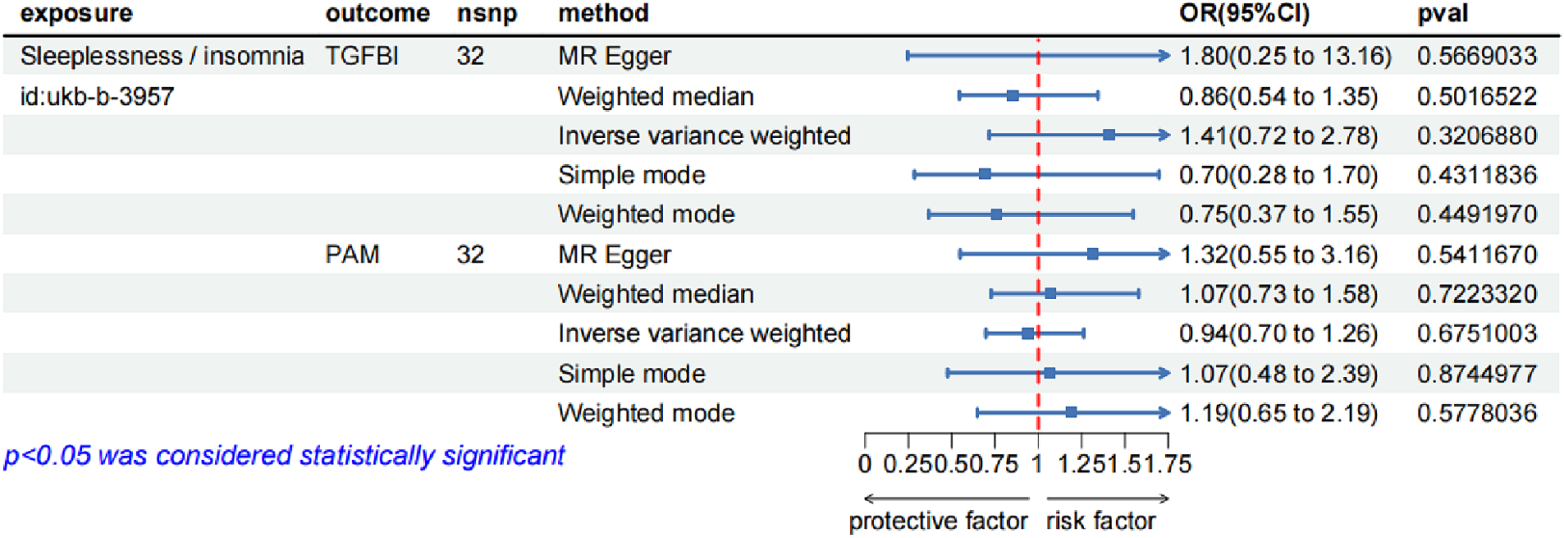
The forest diagram of the reverse MR analysis.

**Figure 5:**
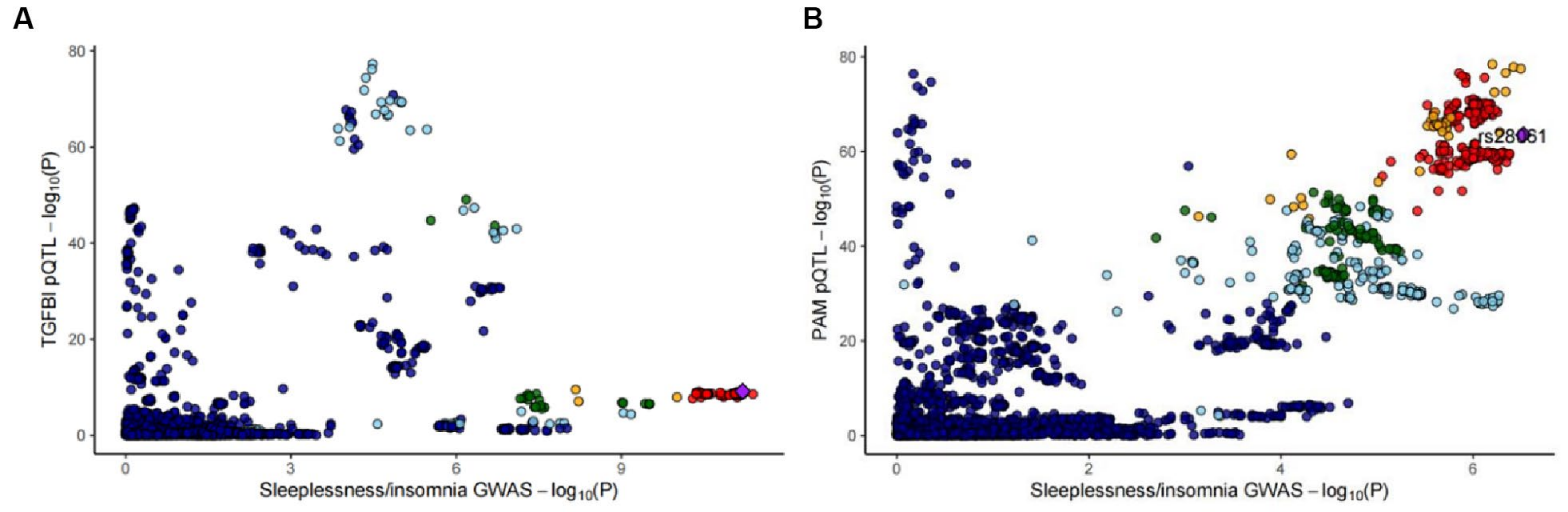
Bayesian co-localization analysis of TGFBI **(A)** and PAM **(B)** with insomnia.

In the reverse MR analysis, we screened 42 SNPs from the insomnia dataset (id = ukb-b-3957) as the exposures based on the same criteria. Supplementary Table S5 lists the detailed information on the 42 SNPs. We then extracted the SNPs of TGFBI and PAM from the original research study 1 as the outcome. The reverse MR analysis indicated no causal effects of insomnia on the protein levels of TGFBI and PAM (Figure 4), and the results of Steiger filtering further ensured the directionality of our findings. Furthermore, the Bayesian co-localization analysis manifested that PAM (coloc.abf-PPH4 = 0.823) shared the same genetic variant with insomnia, while the co-localization result of TGFBI with insomnia was negative. The visualization image of Bayesian co-localization analysis is shown in Figure 5. Phenotype scanning revealed that PAM (rs257309) was connected with the clinical feature of “nap during day,” whereas no significant associations were observed for TGFBI. The details of the sensitivity analysis are listed in Table 2.

**Table 2:** Summary of sensitivity analysis of TGFBI and PAM.

Protein	UniProt ID	SNP	Reverse MR (MR-IVW)	Steiger filtering	Co-localization PPH4 (coloc.abf)	Previously reported
TGFBI	A0A0S2Z4Q2;Q15582	rs13159365	1.41 (0.72–2.78)	TRUE 6.41E-72	2.77E-06	None
PAM	P19021;O43832	rs257309	0.94 (0.70–1.26)	TRUE 2.02E-72	0.823	Nap during day

### 3.3 PPI network analysis

**Figure 6:**
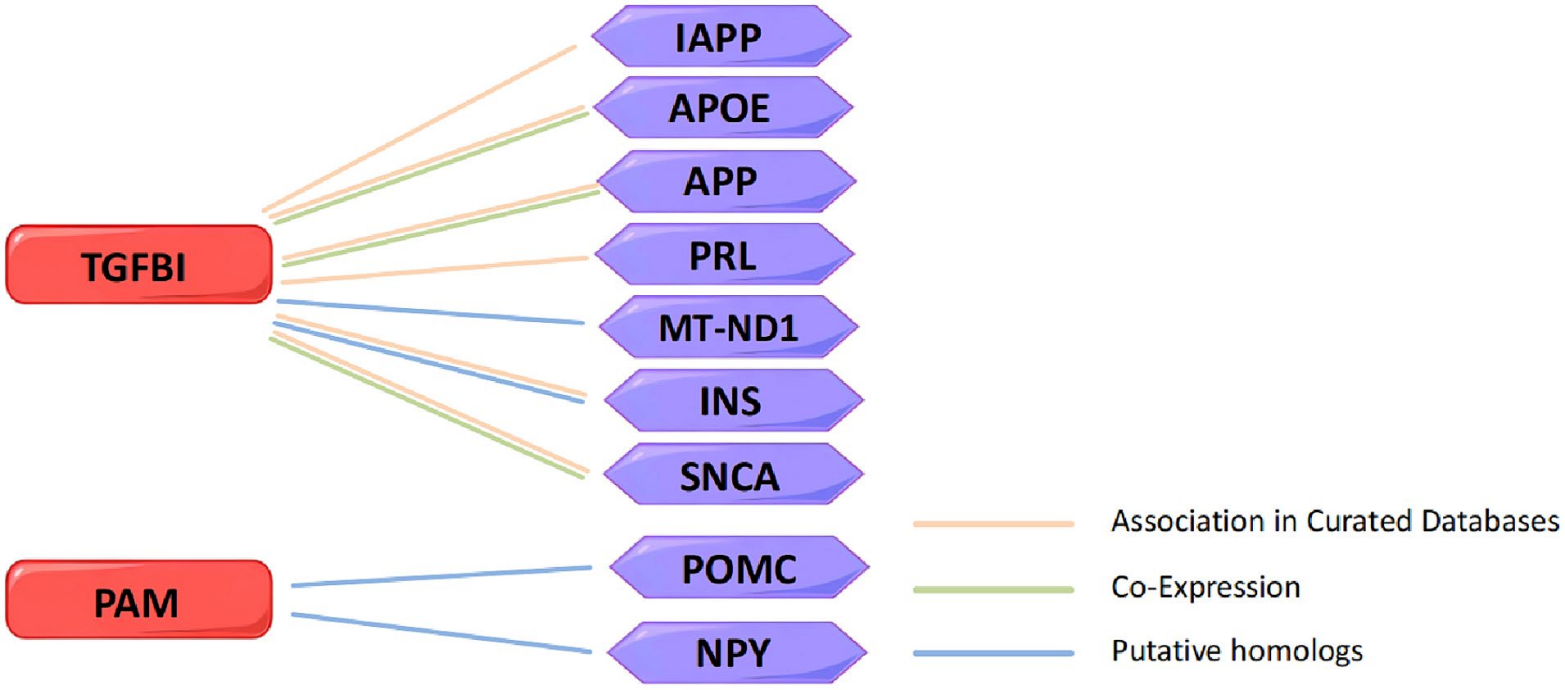
The currently identified insomnia targets related to TGFBI and PAM in the PPI network.

In order to explore the interaction between TGFBI and PAM with marketed drug targets, we screened 137 drug targets for treating insomnia from the DrugBank and the Open Targets databases and attempted to construct a PPT network to study their interaction relationships. Unfortunately, we did not identify any association between TGFBI and PAM with the marketed drug targets (Supplementary Figure S1). Because of this, we searched for insomnia-related targets in the GeneCards database and established another PPI network of TGFBI, PAM, and the top 335 targets with correlation scores greater than 1.5, and found some interaction relationships (Supplementary Figure S2). STRING revealed that TGFBI is related to islet amyloid polypeptide (IAPP), apolipoprotein E (APOE), amyloid beta precursor protein (APP), prolactin (PRL), mitochondrially encoded NADH dehydrogenase 1 (MT-ND1), insulin (INS), and synuclein alpha (SNCA). Among them, TGFBI has gene co-expression with APP, APOE, and SNCA. TGFBI and MT-ND1 interacted physically, indicating that they are in close proximity. PAM is associated with proopiomelanocortin (POMC) and neuropeptide Y (NPY). The currently identified insomnia targets related to TGFBI and PAM in the PPI network are shown in Figure 6.

## 4 Discussion

The development of human proteomics provides a crucial foundation for drug research. Therefore, in order to discover potential new therapeutic targets for insomnia, we combined MR and co-localization approaches to evaluate proteins with causal effects on insomnia, and clinically translated the findings from previous GWAS studies. Initially, MR analysis to preliminarily identify the protein targets TGFBI and PAM as potential pathogenic factors contributing to insomnia. Subsequently, we substantiated these findings through external validation. Additionally, the co-localization analysis provided evidence of gene-based co-localization between PAM and insomnia. To gain insights into the potential mechanisms underlying the association of TGFBI and PAM with insomnia, we endeavored to construct a PPI network to facilitate our comprehension of the underlying pathways involved.

TGFBI, previously also known as βig-h3 and BIGH3, is a gene that encodes a protein in the extracellular matrix of corneal epithelial cells. Previous studies have observed elevated expression levels of TGFBI in active growth regions such as skin, bone, kidney, and horn (26). It plays an important role in hereditary corneal dystrophy (27), epidermal stem cell proliferation and re-epithelization (28), diabetes (29), and tumor progression (30). The relationship between TGFBI and insomnia was first reported through a genome-wide association analysis of sleep disorder characteristics (31), which identified TGFBI as a locus for female insomnia symptoms. Although the underlying mechanism between TGFBI and insomnia remains unknown, we tried to identify potential associations through the discovery of PPI networks. In the study of Alzheimer’s disease, it was discovered that although APP itself is a target of miRNA regulation, amyloid β protein can downregulate miRNAs, including miR-181c and miR-9, both of which inhibit the expression of TGFBI (32). Additionally, studies have found elevated levels of amyloid beta in individuals with insomnia (33). Based on these results, we hypothesize that TGFBI may contribute to insomnia in conjunction with the regulation of APP and miRNAs, among other factors. Furthermore, research has confirmed that insufficient sleep can lead to an increase in insulin resistance (34, 35), and a recent study has shown that TGFBI-deficient mice exhibit resistance to insulin resistance (36). These findings provide further support for our hypothesis regarding TGFBI’s role as a pathogenic factor in insomnia. SNCA mutation carriers are mostly associated with sleep disorders (37) and genes involved in TGF-β signaling and apoptosis pathways are significantly up-regulated in SNCA-deficient mice (38). Thus, we speculate that TGFBI, as a TGF-β-induced protein, may also contribute to insomnia through these pathways.

Numerous neuronal and endocrine processes rely on amidated peptides, while, PAM is the only bifunctional enzyme for amidated peptide biosynthesis (39). Although MR and co-localization analysis support that PAM increases the risk of insomnia, there are currently no clinical reports or studies exploring the underlying mechanisms. However, studies have demonstrated that PAM catalyzes the formation of oleamide from oleoylglycine *in vitro*, which has sleep-restoring properties (40). In the PPI network, we found that PAM has an associative effect with POMC and NPY. PAM can catalyze the conversion of modified POMC to proadrenocorticotropic hormone and then to alpha-melanocyte stimulating hormone (41). Intravenous injection of POMC-derived peptides had varying effects on sleep-wakefulness. For instance, norepinephrine (1 μg) had an arousal effect, while desmoplasmin (1 ng) and norepinephrine-like intermediate lobe peptide (10 ng) increased slow-wave sleep and paradoxical sleep, respectively (42). NPY promotes sleep (43), and PAM serves as a rate-limiting enzyme in neuropeptide maturation. It has been shown that primary insomnia patients in China have significantly lower morning plasma NPY levels compared to normal controls (8). We speculate that this may be attributed to a series of catalytic products mediated by PAM, such as NPY and POMC derivatives, which collectively impact sleep. The underlying mechanisms likely involve a series of interconnected reactions.

Our research has the following advantages. Firstly, the integration of GWAS and pQTL data in MR can help identify drug targets, reduce experimental biases, and minimize confounding factors, thereby improving the success rate of drug development (14). Given that causality identified by MR may be influenced by various confounding factors such as reverse causality, horizontal pleiotropy, and LD-related genetic confounding (12), we performed a set of sensitivity analyses to account for their potential impact. Bidirectional MR analysis demonstrated that the identified proteins did not exhibit a reverse causal relationship, which was further validated through Steiger filtering. To minimize the impact of horizontal pleiotropy, we solely selected cis-pQTLs as instrumental variables, as they directly affect the transcription and/or translation of related genes (44). Furthermore, Bayesian co-localization was employed to mitigate any bias introduced by LD. Phenotype scanning effectively ruled out the presence of horizontal pleiotropy and revealed a correlation between PAM and the clinical characteristics of daytime napping, a common symptom of insomnia. This finding further supports our research findings to a certain extent.

Our study also has some limitations. Firstly, the proteins included in our study were sourced from various studies. Although the GWAS of the circulating protein data from four studies (17, 19, 21, 22) were based on aptamers, it is still possible that biases exist due to inconsistent measurements across these studies. Secondly, there is a limited number of SNPs with two or more in the exposed protein summary data, and TGFBI and PAM each has only one cis-acting SNP. This restricted our ability to conduct a comprehensive analysis based on the preliminary results. However, a series of sensitivity analyses were performed to minimize any biases in our findings. In addition, our analysis only focused on European individuals, so careful consideration should be given when extrapolating these findings to different populations. Lastly, despite our efforts, we did not identify any drug targets associated with TGFBI and PAM in the current treatment of insomnia. Perhaps our results may bring about new insights into the mechanisms of insomnia. The results of the PPI analysis are only suggestive and further research is needed to uncover the underlying mechanisms involved. These limitations should be acknowledged and addressed in future studies to strengthen the validity and generalizability of our findings.

## 5 Conclusion

Our integrative analyses indicate that there is a causal connection between genetically determined levels of circulating TGFBI and PAM and the risk of insomnia, which may serve as appealing drug targets for treating insomnia, especially PAM. Further researches are needed to detect the potential mechanisms of TGFBI and PAM in insomnia.

## Data availability statement

The datasets presented in this study can be found in online repositories. The names of the repository/repositories and accession number(s) can be found in the article/Supplementary material.

## Ethics statement

Ethical approval was not required for the studies involving humans because all data in our study were acquired from publicly available datasets. Ethical approval was obtained for each cohort, and informed consent was derived from all enrollments before their involvement. The studies were conducted in accordance with the local legislation and institutional requirements. The human samples used in this study were acquired from gifted from another research group. Written informed consent to participate in this study was not required from the participants or the participants’ legal guardians/next of kin in accordance with the national legislation and the institutional requirements.

## Author contributions

NY: Conceptualization, Formal analysis, Methodology, Writing – original draft. LS: Validation, Writing – review & editing. PX: Validation, Writing – review & editing. FR: Supervision, Writing – review & editing. SL: Validation, Writing – review & editing. CL: Supervision, Writing – review & editing. XQ: Supervision, Writing – review & editing.
