# Supplementary material for: Identification of potential drug targets for insomnia by Mendelian randomization analysis based on plasma proteomics
Source: Front Neurol. 2024 Apr 25;15:1380321. doi: 10.3389/fneur.2024.1380321 (PMC11079244; doi:10.3389/fneur.2024.1380321)

Supplementary Material

# Supplementary Tables

The Supplementary Tables can be found in the file "Supplementary Tables. xlsx", which includes the following tables:

Supplementary Table 1. The databases and analysis websites involved in this study.

Supplementary Table 2. Genetic instruments of plasma for MR analysis.

Supplementary Table 3. Heterogeneity analysis on proteins with two or more instruments.

Supplementary Table 4. Genetic instruments of potential causal proteins for external validation.

Supplementary Table 5. Genetic instruments of insomnia for reverse MR.

# Supplementary Figures

**Supplementary Figure 1.** Supplementary Figure 1. The PPI network of the identified targets of insomnia with TGFBI and PAM.
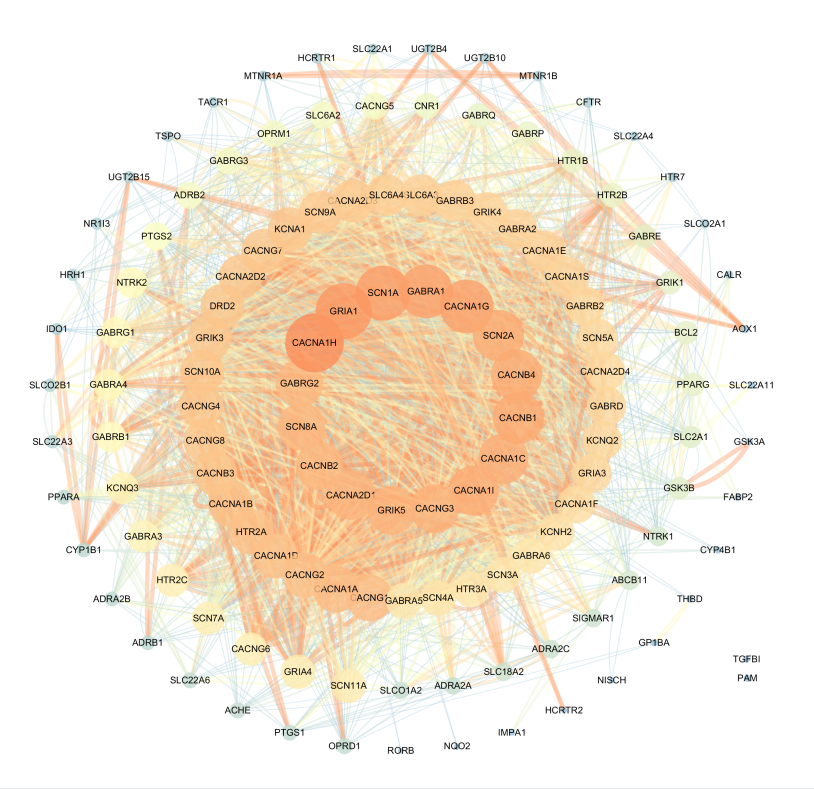


**Supplementary Figure 2.** The PPI network of the top 335 targets of insomnia with TGFBI and PAM.


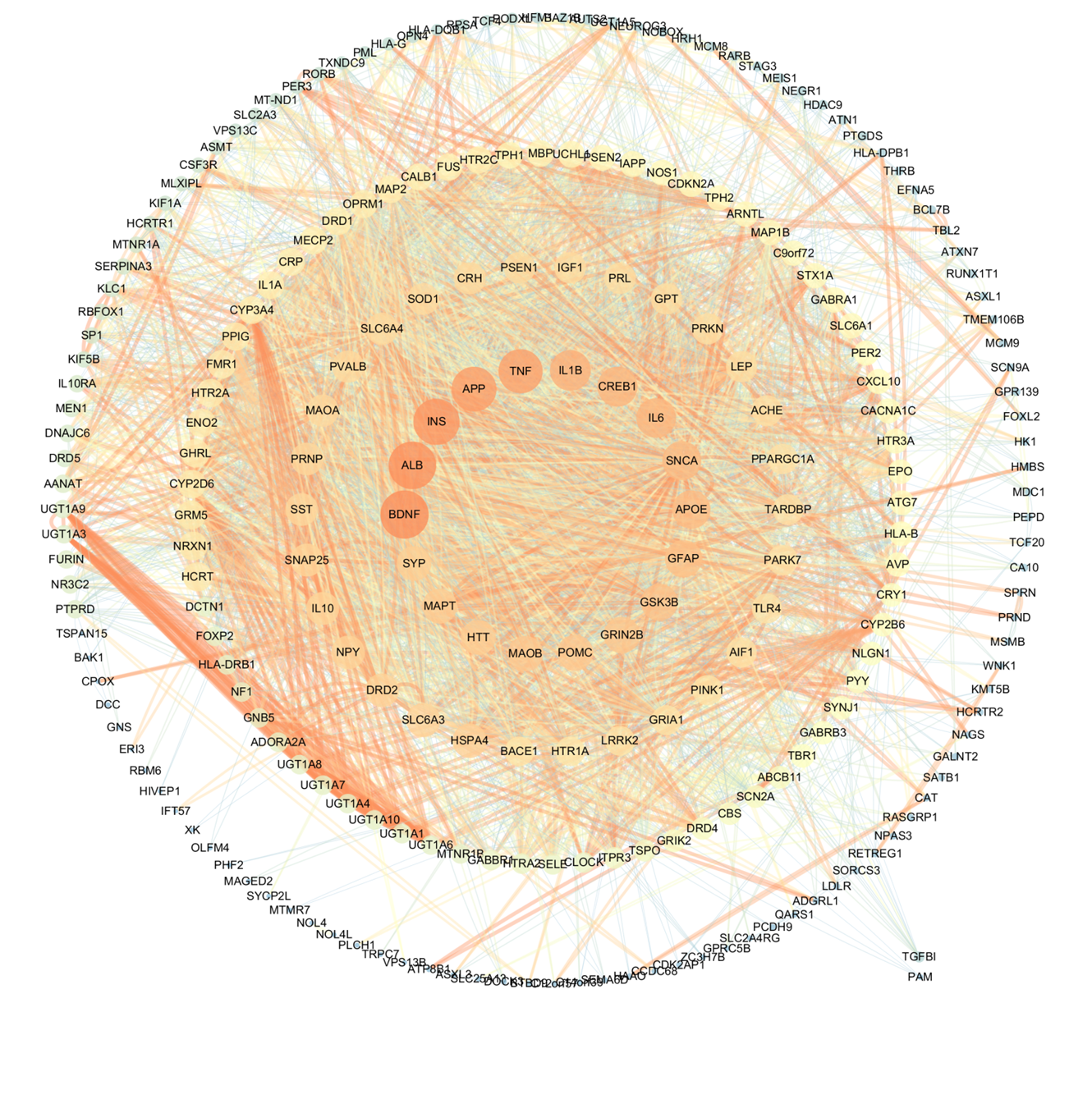

Supplement: Supplementary file 1 [file Data_Sheet_1.DOCX]
